# Effects of Partially Hydrolyzed Guar Gum Supplementation on the Fecal Microbiotas of Piglets

**DOI:** 10.3390/pathogens10111420

**Published:** 2021-11-01

**Authors:** Ryo Inoue, Hikari Otabi, Taiga Yamashita, Naoya Takizawa, Toshinobu Kido, Akira Sugiyama, Makoto Ozeki, Aya Abe, Takamitsu Tsukahara

**Affiliations:** 1 Laboratory of Animal Science, Department of Applied Biological Sciences, Faculty of Agriculture, Setsunan University, Nagaotoge-cho 45-1, Osaka 573-0101, Japan; hikari.otabi@setsunan.ac.jp (H.O.); taiga.yamashita0213@gmail.com (T.Y.); 2 Osato Livestock Industry Co., Ltd., Tsu 514-0126, Japan; n_takizawa@crestfarm.co.jp (N.T.); kido@oosatofarm.co.jp (T.K.); a-sugiyama@oosatofarm.co.jp (A.S.); 3 Nutrition Division, Taiyo Kagaku Co., Ltd., Yokkaichi 510-0844, Japan; mozeki@taiyokagaku.co.jp (M.O.); aabe@taiyokagaku.co.jp (A.A.); 4 Kyoto Institute of Nutrition & Pathology, Ujitawara, Kyoto 610-0231, Japan; tsukahara@kyoto-inp.co.jp

**Keywords:** pig, gut microbiota, dietary fiber, prebiotics

## Abstract

Probiotics and prebiotics have become viable alternatives of growth-promoting antimicrobials in animal production. Here, we tested partially hydrolyzed guar gum (PHGG) as a possible prebiotic for piglets in the commercial farm. Five hundred and ninety-four piglets were used for the experiments, with 293 given a normal pig feed (control), while the rest the feed plus 0.06% (w/w) of PHGG (PHGG). One and three months post-PHGG supplementation, fecal samples were collected from randomly selected 20 piglets in each group and analyzed for microbiota and organic acid concentrations. Notably, the abundance of *Streptococcus,* and unclassified *Ruminococcaceae* were lower (*p* < 0.05) in PHGG than in control, one-month post-supplementation. *Lactobacillus* and *Prevotella* were higher (*p* < 0.05), while *Streptococcus* was lower (*p* < 0.05), in PHGG than in control, three months post-supplementation. The concentrations of acetate, propionate, and butyrate were greater in PHGG than in control, three months post-supplementation. Finally, PHGG grew faster and had fewer deaths until slaughter time (*p* < 0.05), than control. We concluded that PHGG not only was an effective prebiotic to alter gut microbiota of weanling piglets but also can possibly promote body weight accretion and health.

## 1. Introduction

The gastrointestinal tract of the piglet is rapidly populated with a complex and diverse microbial community immediately after birth [1]. However, in modern pig industry, the weaning of piglets usually takes place at 3–4 weeks of age [2], abruptly exposing piglets to a myriad of new environmental, psychological, and nutritional events [3]. Therefore, gastrointestinal disorders are commonly observed immediately post-weaning [4]. 

It has been shown that lactic acid bacteria, enterobacteria, and streptococci are the major first colonizers of the piglet’s intestine, but lactobacilli numbers drop significantly after weaning [5], while others such as Proteobacteria and Bacteroidetes increase [6]. Adhikari et al. observed that piglets suffering from postweaning diarrhea had a high abundance of *Campylobacter* in their intestinal samples. Similarly, McCormack et al. [7] reported that piglets with low feed efficiency had a high abundance of *Streptococcus* spp. in the intestinal and fecal samples, 42 days postweaning. Conversely, polysaccharide-degrading *Prevotella* increases after weaning [8]. As the efficiency by which the animal utilizes feed to reach the optimal body weight by the required finishing time, determines production performance [9], a healthy microbiota is deemed necessary to maintain the homeostasis and hence production performance of the piglet. 

Antimicrobials have been used to control infections by pathogenic microorganisms [10] and promote growth in farm animals, pigs included [11]. However, global concern regarding the emergence of drug-resistant microorganisms has prompted the ban of antimicrobials as growth promoters [10,12]. Probiotics, i.e., a broad term to define those microorganisms that confer health benefits to the host when consumed regularly, have been favored as alternative growth promoters [12]. Probiotics putatively colonize the gastrointestinal tract of piglets and beneficially modify the microbiota by competing with and outnumbering pathogenic or opportunistic strains [13,14]. To promote pig growth the industry has started adding prebiotics, which serve as substrate for probiotics and beneficial bacteria in the microbiota [13,14]. Supplementation with prebiotics galacto-oligosaccharide and oligofructose-enriched inulin improved the weight gain of weaned piglets challenged with enterotoxigenic *Escherichia coli* [15] and lowered the pathogen’s colonization in *Salmonella* Typhimurium-infected piglets [16], respectively, possibly by promoting the growth of indigenous beneficial microorganisms. Fu et al. [17] incubated solutions prepared from porcine fecal samples with fermented and hydrolyzed derivatives of guar gum processed from seed endosperm of *Cyamopsis tetragonoloba*. They reported that high concentrations of lactate, acetate, and propionate were produced when hydrolyzed guar gum was added to the incubated solutions. In addition, increases in the abundances of *Clostridium sensu stricto* 1 and *Bifidobacterium* were observed when hydrolyzed guar gum was added to the incubated solution [17]. 

Mudgil et al [18] touted partially hydrolyzed guar gum (PHGG), a soluble fiber from guar gum, as a prebiotic with high potential. They showed that PHGG promoted the growth of lactic acid bacteria in vitro. More recently, we showed at these premises that children with autism spectrum disorders and supplemented with PHGG experienced less constipation and had lower inflammatory cytokines in the blood serum, possibly due to beneficial changes within gut microbial communities [19] promoted by PHGG supplementation. We theorized that supplementation of PHGG to piglets may confer similar health benefits to piglets undergoing weaning stress. Therefore, in the present work, to elucidate the possible prebiotic effects of PHGG on the microbiota of weaned piglets, we added it to their diets. In addition, to evaluate the metabolism of dietary fiber, the concentrations of organic acids were measured.

## 2. Results

### 2.1. Alpha Diversity of Chao1 and Shannon Indices in the Fecal Samples of Piglets

**Figure 1:**
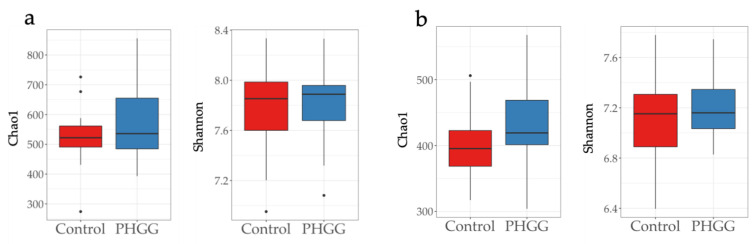
Chao1 and Shannon indices in the fecal microbiotas of piglets, (**a**) one and (**b**) three months post-supplementation of partially hydrolyzed guar gum (PHGG). *n* = 20.

In total 2,273,490 high quality sequence reads (28,419 ± 6973 reads per sample) were obtained after quality filtering and denosing in this study. There was no significant difference between the control and PHGG groups in both the Chao1 and Shannon indices for α-diversity after one and three months of the addition of PHGG to the diet (Figure 1).

### 2.2. Beta Diversity in the Fecal Samples of Piglets

**Figure 2:**
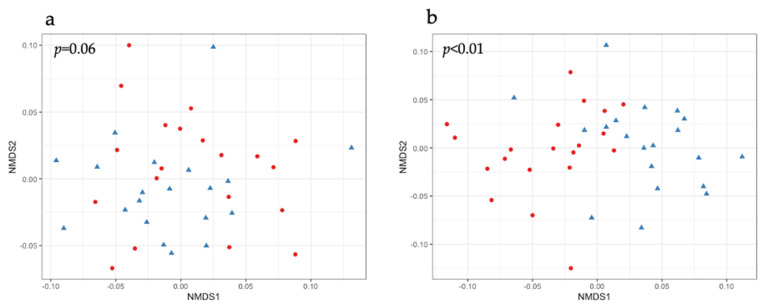
Non-metric multidimensional scaling (NMDS) plots of the fecal microbiotas of piglets, based on weighted UniFrac distances, (**a**) one and (**b**) three months post-supplementation of PHGG. Red circles and blue triangles denote control group and PHGG group, respectively. The difference between groups were significant if *p* < 0.05 by PERMANOVA analysis.

Regarding the β-diversity, one-month post-PHGG supplementation, the fecal microbiota composition of experimental groups only tended to be different when measuring the weighted UniFrac distances (Figure 2a). However, three months post-PHGG supplementation, the compositions of the fecal microbiota of the experimental groups were significantly different (Figure 2b).

### 2.3. Bacterial Composition of Fecal Microbiota

**Figure 3:**
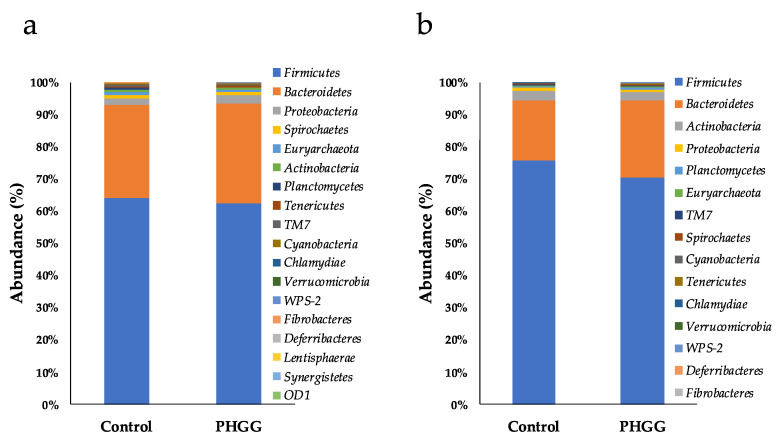
Mean composition of bacterial phyla in the fecal microbiotas of piglets, (**a**) one and (**b**) three months post-supplementation of PHGG. *n* = 20.

Statistically, at phylum level, there were no significant differences in the bacterial abundances between the two groups one-month post-PHGG supplementation (Figure 3a). Conversely, three months post-PHGG supplementation, the abundance of Bacteroidetes was significantly higher in the PHGG group (24.06 ± 2.71%) than in the control group (18.49 ± 4.12%) (*p* < 0.01). In addition, the abundance of Tenericutes was also higher in the PHGG group (0.20 ± 0.13%) than in the control group (0.10 ± 0.07%) (*p* < 0.01) (Figure 3b). Moreover, although in the control group no abundance of Fibrobacteres was detected, they were found at 0.01 ± 0.02% in the PHGG group; the frequency was significantly higher in PHGG group (*p* < 0.05 by chi-square test). By contrast, the abundance of Firmicutes was significantly lower (*p* < 0.01) in the PHGG group (70.37 ± 3.37%) than in the control group (75.81 ± 3.78%) (Figure 3b).

At genus level, there were notable bacteria in the fecal samples of piglets whose abundances differed more than 1.0% between groups. For example, the abundances of genus *Streptococcus* and an unclassified genus of family *Ruminococcaceae* were lower (*p* < 0.05) in the PHGG group (1.60 ± 0.98 and 9.01 ± 2.64%, respectively) than in the control group (4.63 ± 2.87 and 11.03 ± 2.52%, respectively), one-month post-PHGG supplementation (Table 1). In contrast, while the abundances of genera *Lactobacillus* and *Prevotella* were higher (*p* < 0.05) in the PHGG group (15.21 ± 7.15 and 13.97 ± 3.51%, respectively) than in the control group (7.47 ± 5.13 and 11.15 ± 3.92%, respectively), genera *Streptococcus* had lower (*p* < 0.05) abundances in the PHGG group (5.08 ± 3.56%) than in the control group (12.13 ± 3.49%), three months post-PHGG supplementation (Table 1). Other genera whose abundances significantly changed between the experimental groups, both one and three months post-PHGG supplementation, are shown in Supplementary Table S1.

### 2.4. Concentrations of Organic Acids in the Fecal Samples of Piglets

When the concentrations of organic acids were analyzed in the fecal samples of piglets, it was found that one-month post-PHGG supplementation, that of lactate significantly (*p* < 0.01) decreased in the PHGG group (1.24 ± 1.16 mM) when compared with that found in control piglets (3.26 ± 2.68 mM) (Table 2). Conversely, three months post-PHGG supplementation, except for lactate and formate, all organic acids either tended to increase or significantly (*p* < 0.01) increased in the fecal samples of the PHGG group, when compared with those of the control piglets. As for the total concentration of organic acids, while it remained unaltered between the experimental groups one-month post-PHGG supplementation, it was significantly (*p* < 0.01) higher in the PHGG group (251.65 ± 31.32 mM) than in the control group (213.55 ± 35.83 mM), three months post-PHGG supplementation (Table 2).

### 2.5. Body Weight at Slaughter and Mortality Rate

It was found that piglets in the PHGG group delivered to slaughter eleven days earlier than those in the control group (158.6 ± 7.7 vs. 169.6 ± 4.1 days, *p* < 0.01). On the other hand, the carcass weight was not significantly different between groups (control group: 76.1 ± 3.7 kg vs. PHGG group: 75.5 ± 4.1 kg). 

The number of piglets dead until slaughter was 30 in the control group, while it was 16 in the PHGG group. The mortality percentage was significantly lower in the PHGG group than in the control group (5.3 vs. 10.3%, *p* < 0.05). 

## 3. Discussion

In commercial animal production, the administration of antimicrobials was the means to promote growth in livestock. However, as mounting evidence has shown that the overuse of antimicrobials has created drug-resistant microorganisms, probiotics are now being used as alternative growth promoters, and prebiotics are being added to feed to maximize the proliferation of both exogenous (probiotics) and endogenous (indigenous microbiota) microorganisms. In the present work, we wanted to investigate whether PHGG supplementation would exert such prebiotic effects on the microbiota present in the guts of piglets.

It has been observed that the gut microbiota thrives on fiber-rich diets, and thus the addition of fiber (including prebiotics) to diets helps increase bacterial numbers and diversity [20]. In the present study, while α-diversity did not significantly differ between experimental groups throughout the study, β-diversity of the fecal microbiota started to differ between experimental groups, being more pronounced at 3 months post-supplementation, in the PHGG group. In humans, PHGG supplementation has been previously shown to cause changes in the β-diversity of the microbiotas in fecal samples from healthy individuals [21]. In pigs, array prebiotics have been found to affect the β-diversity of the colonic microbiota [14]. Based on this evidence, it can infer that in the present work, PHGG supplementation affects the composition of gut microbiota in weanling piglets.

In piglets’ feces, it has been found that the addition of fiber to diets changes the abundance of certain bacteria in the microbiota. For example, the addition of 1% insoluble fiber to piglets’ diets helped increase the abundance of phylum Bacteroidetes, as well as genera *Prevotella*, and of Bacteroidetes, when 0.5% soluble fiber was added as supplement [22]. In adenine-induced chronic kidney disease rats, supplementation of gum acacia helped increase the abundance of phylum Tenericutes [23]. Interestingly, an increase in the abundance of Tenericutes seems to correlate with crude fiber digestibility of pigs [24]. Moreover, in piglets, the supplementation of 5% corn bran and 5% wheat bran caused increases in the abundances of Firmicutes and Fibrobacteres, respectively [25]. Fibrobacteres is the phylum that includes genus *Fibrobacter*, which is a well-known fiber-degrading bacteria [26]. In the present study, Tenericutes and Fibrobacteres abundance was found to be higher in PHGG group than control group 3 months post-PHGG supplementation, which seemed to be because PHGG acted as fiber substrate for these bacteria. Similarly, Le Sciellour et al. found family *Ruminococcaceae* was negatively correlated with fiber digestibility in pigs [27]. A decrease in unclassified genus of family *Ruminococcaceae* 1 month post-supplementation may be due to a similar reason with the increase in phylum Tenericutes and Fibrobacteres.

In view of genus *Lactobacillus*, in weaned piglets, lactobacilli increased in their fecal microbiota when their diets were supplemented with soluble fiber, even when they were experimentally challenged with enterotoxigenic *Escherichia coli*, which seems to imply that the microbiota was more resilient against pathogenic colonization when fiber was added to the diet compared to than if it was not [28]. Lactobacilli are one of the abundant bacteria in the pig gut [29] and, as aforementioned, thrive when diets contain soluble fiber. Therefore, PHGG supplementation seemed to have promoted the proliferation of beneficial Firmicutes such as lactobacilli, while suppressing the growth of less desirable genera such as *Streptococcus*, whose abundances are usually associated with disease [30].

In the present study, the total concentration of organic acids increased in the fecal samples of the PHGG group than in the control group when it was measured 3 months post-PHGG supplementation (Table 2). Soluble fiber was previously found to help increase the total concentration of organic acids in a piglet model [22], which is in accordance with our results. From a health standpoint, organic acids such as short chain fatty acids [31] are of special interest, because they induce tissue proliferation, help absorb minerals and fluids while at the same time helping prevent colonic diseases and accumulation of blood cholesterol [32], among other health benefits. In the present work, short chain fatty acids acetate, propionate, butyrate, and valerate increased in fecal samples of the PHGG group but not in those of the control group, three months post-PHGG supplementation. In the case of valerate, although there is some evidence suggesting that it plays a role in controlling the growth of opportunistic pathogens such as *Clostridioides difficile* [33], its biological functions remain to be fully accounted for. In addition, higher productions of acetate, propionate, and butyrate have been shown to contribute to the improvement of the gut barrier [22].

In addition to these results, the facts that PHGG-supplemented were piglets delivered to slaughter eleven days earlier than control piglets, while the carcass weight did not significantly differ between groups, seemed to indicate that PHGG-supplemented piglets were growing at faster rates, due to their microbiotas being healthy and thriving. The fewer deaths in PHGG group than control group also implied healthier gut microbiotas in PHGG-supplemented piglets. Many correlative studies have indicated the positive association of genus *Prevotella* and negative association of genus *Streptococcus* with growth performance such as body weight and average daily gain [34]. Thus, reaching the desired body weight in PHGG group, in which abundance of genus *Prevotella* and *Streptococcus* were higher and lower, respectively than control group 3 months post-supplementation, was in accordance with these studies. In view of genus *Prevotella* 3 months post-supplementation, the difference in mean relative abundance between groups was 2.82%, while that in genus *Lactobacillus* and *Streptococcus* was more than 7%. In the previous work regarding PHGG supplementation to children with autism spectrum disorder, the difference observed in the bacterial genera between pre- and post-supplementation was 2.34% at maximum, while significant improvement of constipation, systemic inflammation and behavioral irritability was confirmed [19]. Therefore, even the difference in genus *Prevotella* between groups seemed to be small, it could have impact, at least some extent, on health of piglets. 

## 4. Materials and Methods

### 4.1. Animals and Diets

A total of 594 piglets (Large White × Landrace × Duroc; age at 21 days old) were raised in a commercial farm in Mie Prefecture. Of these, 292 piglets were allocated to the control group and 302 to the PHGG group. The experimental groups were kept in different facilities until delivery to slaughterhouse. As it was difficult to evaluate the control group and the PHGG group at the same time, the piglets weaned one week after the control group were designated as the PHGG group. The control group was fed a commercial feed (Chubushiryo Co Ltd., Aichi, Japan), and the PHGG group was fed the same feed as the control group and supplemented with 0.06% (w/w) of PHGG (Sunfiber®; Taiyo Kagaku Co. Ltd., Mie, Japan) without interruption from weaning to finishing. The concentration of PHGG to be supplemented was evaluated by preliminary experiment; four sows fed 0.02, 0.06, and 0.3% PHGG-supplemented diet for a week. As a result, 0.06% was found to be the minimum concentration that significant change in fecal microbiota and organic acid concentration was observed.

### 4.2. Fecal Sampling

Piglet feces were collected one (piglet age: 43–51 days old) and three (average piglet age: 101–108 days old) months after the start of PHGG supplementation. Feces were collected immediately after defecation in such manner that they were free from contamination from the surroundings (dust, etc.), and stored at −20 °C or below until further use. For the experiment, feces were collected from 20 pigs randomly selected from each group, for a total of 40 piglets from each group.

### 4.3. DNA Analysis

Bacterial DNA was extracted from piglet feces using QuickGene DNA tissue kit S (KURABO, Osaka, Japan) as per written in previous study [35] to analyze the intestinal microbiota by 16S rRNA metagenomic analysis.

A library was prepared for 16S rRNA metagenomic analysis according to Inoue, et al. [36]. After that, it was applied to the reagent cartridge of MiSeq Reagent Kit V3 600PE (Illumina, San Diego, CA, USA) and sequenced by MiSeq platform (Illumina, San Diego, CA, USA). Sequence data were analyzed using QIIME2 (ver. 2020.8) [37]. Briefly, the sequence was first denoised by DADA2 plugin of QIIME2 with trimming length from the left set at 17 and from the right at 19. The Sklearn classifier was used for taxonomic assignment against the Greengenes database (13_8; 99% OTUs full-length sequence). In this study, singletons and ASVs (amplicon sequence variants) assigned to mitochondria and chloroplasts were removed. The phylogenetic tree was generated by SATé-enabled phylogenetic placement (SEPP) [38]. The metrics for alpha- and beta-diversities were calculated by QIIME2 by setting the sampling depth at 5000.

### 4.4. Analysis of the Concentrations of Organic Acids

The organic acid concentration in pig feces was measured by ion exclusion high performance liquid chromatography as described in Tsukahara, et al. [39].

### 4.5. Body Weights at Slaughter and Mortality Rates of Piglets

The age, the mortality rates, and carcass weight of piglets at slaughter were obtained from the farms’ data. Incidentally, the farms’ data showed that piglets were generally delivered to slaughterhouses twice per week.

### 4.6. Statistical Analysis

The Chao1 index (species richness) and Shannon index (species evenness) of α-diversity were calculated by QIIME2 [37]. β-diversity was calculated based on UniFrac distance using QIIME2 and visualized by NMDS using the phyloseq package of R statistical analysis software. UniFrac distances between samples were analyzed by the permutational multivariate analysis of variance (PERMANOVA) in QIIME2 [37]. Bacterial abundance at phylum level (%) was compared by Welch’s *t*-test using STAMP [40] and those at genus level were compared by the Wilcoxon ran-sum test using R software (ver. 4.0.3). The difference of organic acid concentration and carcass weight was analyzed by Welch’s *t*-test, that of α-diversity indices was analyzed by the Wilcoxon rank-sum test using R software. The age at slaughter was also compared by the Wilcoxon rank-sum test and the mortality rate was evaluated by chi-square test by R software. The threshold for significance was set to *p* < 0.05.

## 5. Conclusions

Prebiotic supplementation has become a viable strategy to increase animal production. In the present study, we supplemented piglets with PHGG as a possible prebiotic in pigs. Our results showed that PHGG, acting as a substrate, contributed to a greater proliferation of beneficial bacteria such as *Prevotella* and *Lactobacillus*, three months post-PHGG supplementation. In addition, our DNA analyses showed that the abundances of less desirable bacteria, such as genera *Streptococcus,* decreased during both one and three months post-PHGG supplementation. After three months of PHGG supplementation, the concentrations of health-promoting acetate, propionate, butyrate increased, which was very likely because of the improvement of gut microbiota. Better gut microbiota in PHGG group than control group was supposed to be a reason PHGG-supplemented piglets grew faster and had fewer deaths at slaughter time. To conclude, by the present work, it was shown that PHGG not only was an effective prebiotic to alter gut microbiota of weanling piglets but also can possibly promote body weight accretion and health.

## Figures and Tables

**Table 1:** Bacteria that notably differed in the fecal microbiotas of piglets, one and three months post-supplementation of PHGG.

One Month Post-Supplementation					Control			PHGG			
Phylum	Class	Order	Family	Genus	Abundance (%)			Abundance (%)			*P*-value
Firmicutes	Bacilli	Lactobacillales	*Streptococcaceae*	*Streptococcus*	4.63	±	2.87	1.60	±	0.98	<0.01
					(3.97)			(1.43)			
Firmicutes	Clostridia	Clostridiales	*Ruminococcaceae*	Unclassified	11.03	±	2.52	9.01	±	2.64	0.04
					(11.11)			(9.33)			
Three months post-supplementation											
Bacteroidetes	Bacteroidia	Bacteroidales	*Prevotellaceae*	*Prevotella*	11.15	±	3.92	13.97	±	3.51	0.04
					(10.58)			(13.98)			
Firmicutes	Bacilli	Lactobacillales	*Lactobacillaceae*	*Lactobacillus*	7.47	±	5.13	15.21	±	7.15	<0.01
					(6.82)			(16.77)			
Firmicutes	Bacilli	Lactobacillales	*Streptococcaceae*	*Streptococcus*	12.13	±	3.49	5.08	±	3.56	<0.01
					(11.97)			(4.82)			

Genera of which mean relative abundance differed more than 1% between groups and having significant differences at each time point are listed. Other genera having significant differences between groups are listed in Table S1. Data are expressed as mean ± SD (*n* = 20). Median of relative abundance are shown in parentheses. Data were significant if *p* < 0.05.

**Table 2:** Concentrations of organic acids in the fecal microbiotas of piglets, one and three months post-supplementation of PHGG.

1 Month Post-PHGG Supplementation								3 Months Post-PHGG Supplementation						
mM	Control			PHGG			*p*-Value	Control			PHGG			*p*-Value
Succinate	0.67	±	0.38	0.79	±	0.29	0.25	1.18	±	0.64	1.68	±	0.98	0.07
Lactate	**3.26**	**±**	**2.68**	**1.24**	**±**	**1.16**	**<0.01**	4.05	±	3.56	3.34	±	1.60	0.42
Formate	0.76	±	0.46	0.62	±	0.42	0.32	**0.82**	**±**	**0.44**	**0.58**	**±**	**0.32**	**0.04**
Acetate	132.17	±	15.55	123.14	±	18.70	0.11	**128.45**	**±**	**19.14**	**144.18**	**±**	**17.36**	**<0.01**
Propionate	39.27	±	5.32	40.12	±	9.62	0.73	**48.85**	**±**	**11.41**	**59.39**	**±**	**11.83**	**<0.01**
iso-butyrate	2.34	±	1.10	2.15	±	1.78	0.70	**2.38**	**±**	**0.99**	**3.26**	**±**	**1.07**	**<0.01**
n-butyrate	18.01	±	4.49	21.11	±	9.93	0.21	**20.46**	**±**	**5.70**	**28.24**	**±**	**7.02**	**<0.01**
iso-valerate	2.95	±	1.68	3.37	±	2.74	0.57	**2.81**	**±**	**1.35**	**4.36**	**±**	**1.51**	**<0.01**
n-valerate	2.20	±	1.03	2.93	±	2.10	0.17	**4.56**	**±**	**1.63**	**6.62**	**±**	**1.61**	**<0.01**
Total organic acids	201.62	±	22.40	195.48	±	40.55	0.56	**213.55**	**±**	**35.83**	**251.65**	**±**	**31.32**	**<0.01**

Data are expressed as mean ± SD (*n* = 20). Data with significant difference (*p* < 0.05) are indicated by bold letters.

## Data Availability

The sequence data have been deposited in the NCBI Sequence Read Archive (SRA) under accession number PRJNA759121. Raw data for composition of gut microbiota analysis can be provided upon reasonable request.

## Supplementary Materials

The following are available online at https://www.mdpi.com/article/10.3390/pathogens10111420/s1, Table S1; Genera not listed in Table 1 but having significant differences between groups at each time point.
